# Synergistic Toughening of Epoxy through Layered Poly(ether imide) with Dual-Scale Morphologies

**DOI:** 10.1021/acsami.3c10096

**Published:** 2023-11-02

**Authors:** Ujala Farooq, Ekaterina Sakarinen, Julie Teuwen, René Alderliesten, Clemens Dransfeld

**Affiliations:** † Faculty of Aerospace Engineering, Aerospace Structures and Materials, Delft University of Technology, Kluyverweg 1, HS Delft 2629, The Netherlands; ‡ Institute of Polymer Engineering, FHNW University of Applied Sciences and Arts Northwestern Switzerland CH-5210, Windisch, Switzerland

**Keywords:** epoxy, poly(ether imide) (PEI), interphase formation, morphology, reaction-induced phase separation, fracture toughness, hierarchical toughening

## Abstract

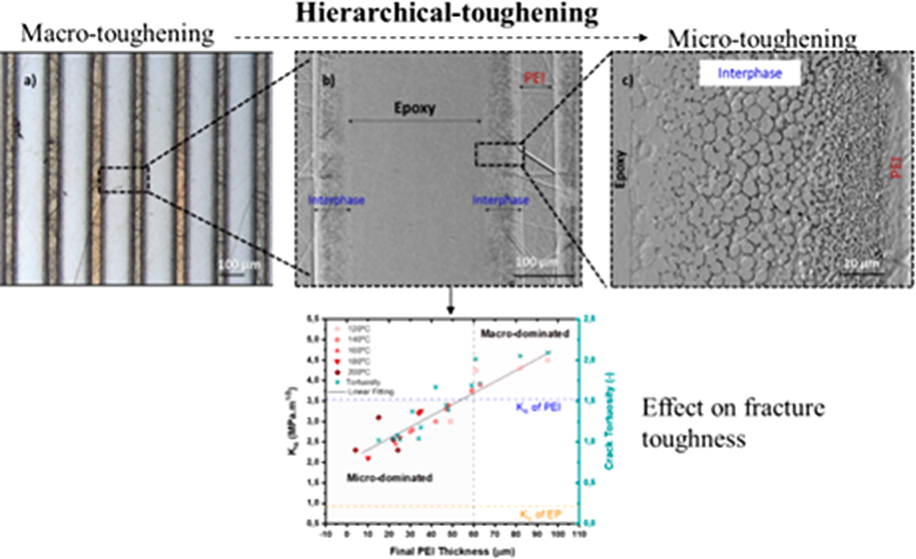

Toughness of epoxies is commonly improved by adding thermoplastic phases, which is achieved through dissolution and phase separation at the microscale. However, little is known about the synergistic effects of toughening phases on multiple scales. Therefore, here, we study the toughening of epoxies with layered poly(ether imide) (PEI) structures at the meso- to macroscale combined with gradient morphologies at the microscale originating from reaction-induced phase separation. Characteristic features of the gradient morphology were controlled by the curing temperature (120–200 °C), while the layered macro structure originates from facile scaffold manufacturing techniques with varying poly(ether imide) layer thicknesses (50–120 μm). The fracture toughness of the modified epoxy system is investigated as a function of varying cure temperature (120–200 °C) and PEI film thickness (50–120 μm). Interestingly, the result shows that the fracture toughness of modified epoxy was mainly controlled by the macroscopic feature, being the final PEI layer thickness, i.e., film thickness remaining after partial dissolution and curing. Remarkably, as the PEI layer thickness exceeds the plastic zone around the crack tip, around 62 μm, the fracture toughness of the dual scale morphology exceeds the property of bulk PEI in addition to a 3 times increase in the property of pure epoxy. On the other hand, when the final PEI thickness was smaller than 62 μm, the fracture toughness of the modified epoxy was lower than pure PEI but still higher than pure epoxy (1.5–2 times) and “bulk toughened” system with the same volume percentage, which indicates the governing mechanism relating to microscale interphase morphology. Interestingly, decreasing the gradient microscale interphase morphology can be used to trigger an alternative failure mode with a higher crack tortuosity. By combining facile scaffold assemblies with reaction-induced phase separation, dual-scale morphologies can be tailored over a wide range, leading to intricate control of fracture mechanisms with a hybrid material exceeding the toughness of the tougher phase.

## 1 Introduction

Epoxy-based composites are subjected to high static and dynamic loadings in engineering applications, which require higher resistance to fracture. In contrast, epoxies with high cross-linking densities are inherently brittle and typically have a low fracture toughness. However, different approaches are known to increase their fracture toughness.^1,2^ Numerous methods have been used to incorporate a second phase into the epoxy matrix, such as rubber, inorganic nanoparticles, or thermoplastics, referred to as bulk resin modification.^3^ These tougheners usually form specific morphologies during the curing phase of epoxy, resulting in an improved fracture toughness of the system. Unfortunately, for some tougheners, the addition of the second phase into the epoxy system also reduces overall modulus and limits the end-use temperature of the system.^4,5^ Moreover, the viscosity of the resin system may also increase significantly by bulk resin modification, primarily through thermoplastics, which makes them unsuitable for liquid composite molding processes.^6^

In contrast to bulk modification, several other approaches and effects to toughen a brittle material have been observed on the macro-, the micro-, and/or the nanoscale.^7,8^ Combining these effects results in hierarchically toughened structures. The first and most relevant approach for toughening on a macro scale is the introduction of ductile interlayers into a brittle material, which leads to toughening through the inhomogeneity effect.^9,10^ The material inhomogeneity effect is based on spatial variations in material properties significantly influencing the crack driving force, i.e., a material inhomogeneity can hinder or promote crack propagation. The ductile interlayers act as crack arresters, so that the fracture toughness of the base material strongly increases. If the ductile interlayers are thin enough, the loss in strength is almost negligible.^11^ For instance, Sistaninia et al. focused on designing new fracture resistance material by utilization of the material based on the yield stress inhomogeneity effect.^12^ They used ductile interlayers (low-strength steel) to toughen the high-strength steel matrix and reported a criterion of optimum architectural parameters, i.e., optimum interlayer spacing. The multilayer specimen’s fracture toughness was at a similar value to the sample having a single interlayer in the case of interlayer spacing larger than the process zone around the crack tip. Likewise, Zechner and Kolednik used aluminum sheets as multilayers with a polymer adhesive.^13^ Structures in different configurations (i.e., crack dividers and crack arresters) are tested. The results showed that the crack arrester configuration exhibits a tremendously improved crack growth resistance (73.7 kJ/m^2^) and fracture initiation toughness compared to the single sheet configuration (33.6 kJ/m^2^), which was attributed to the so-called “material inhomogeneity effect”.^13^ Several other studies introducing soft interlayers into brittle material showed increased fracture toughness due to the inhomogeneity effect.^14,15^ All of these studies highlighted the importance of material inhomogeneity at specific scales in improving the fracture toughness of brittle material. This concept is also adopted in this current study to toughen an epoxy system (brittle material) by using multilayers of poly(ether imide) (PEI, ductile material), leading to the material inhomogeneity effect (i.e., differences in yield stress).

Another potential toughening effect occurs at one scale lower, at the microscale. Several researchers have reported the microtoughening of epoxy using a thermoplastic (PEI).^16−19^ For instance, Harismendy et al., reported the formation of a particulate morphology by adding 10 wt % PEI into the epoxy which resulted in a 2-fold increase in fracture toughness due to crack path deflection mechanism.^16^ Likewise, as reported in our previous study,^20^ in the case of using a thermoplastic film as the ductile interlayer, liquid reactive thermoset monomers diffuse into the glassy thermoplastic and partially swell or dissolve it, which results in the diffusion of TP polymeric chains into the epoxy resin. The mutual diffusion of the components creates a concentration gradient in the interfacial region.^21^ The proceeding cure reaction between the comonomers induces a phase separation leading to a gradient morphology in the interfacial zone.^22^ The gradient interphase shows different morphologies throughout its thickness depending on the concentration of epoxy and PEI. This granular morphology can impart toughening effects similar to those of micro- and nanosized dispersed particles, as found in bulk toughening of thermosetting systems. These “inclusions” can induce various energy dissipation mechanisms, thereby limiting the onset and propagation of cracks by different toughening mechanisms, i.e., crack deflection, crack pinning, or debonding of particles.^23,24^ The cure profile strongly affects the thickness of the interphase and its morphology.^20^

The aim of this study is to understand the effect of micro- and macrotoughening approaches on the fracture toughness of an epoxy system by using thermoplastic PEI multilayers. Thereby, the following research questions are addressed: (i) what are the respective effects of macrotoughening (i.e., submillimeter range) and microtoughening (i.e., micron size range) on the fracture toughness of epoxy? (ii) Is there any synergetic effect between these two toughening mechanisms? From the morphological point of view, it is not known whether a larger scale heterogeneity in the submillimeter range, by having thermoplastic multilayers, would possibly be more effective. Therefore, the first research question is addressed by toughening the epoxy resin using multilayers of PEI with varying thicknesses (50–120 μm) to tune the macrotoughening phenomenon. The second research question is investigated by developing diffusion-controlled interphase gradients (micron-sized morphology) between the aforementioned multilayers at the epoxy/PEI interface as a function of different cure temperatures (120–200 °C) and determining the resultant fracture toughness of the system. We, therefore, present a method to create dual-scale morphologies by (a) assembling multilayer scaffolds of thermoplastic tougheners with architected porosity (giving rise to the morphology in the submillimeter range) and (b) creating diffusion-controlled interphases (micron-sized morphology) along with the reaction-induced phase separation after infiltration with an epoxy system. This allowed us to discriminate the effect of the thickness of the thermoplastic layer, cure temperature, interphase thickness, and morphological features on the fracture toughness of the resultant hybrid material.

## 2 Experimental Section

### 2.1 Materials

Thin films of PEI (ULTEM 1000, molecular weight = 55,000 g/mol, *n* ≈ 90, *T*_g_ = 217 °C), with different thicknesses (50, 60, 90, and 120 μm), provided by SABIC, Saudi Arabia, were used as the tough phase. The thermoset resin was prepared as a blend of M-(2,3-epoxypropoxy)-*N*,*N*-bis(2,3-epoxypropyl)aniline (TGMAP, Araldite MY 0610 CH), and bisphenol-F epoxy resin monomer (DGEBF, Araldite PY 306 CH). 3,3′-sulfonyldianiline (DDS, Aradur 9719-1) was used as a curing agent for epoxy. Huntsman, Switzerland, supplied all these chemicals.

### 2.2 Methodology

#### 2.2.1 Resin Preparation

Epoxy resin was prepared by mixing TGMAP and DGBEF at 78.50 and 21.50 wt %, respectively. The specific amount of curing agent (28.75 g) was then incorporated into 50 g of epoxy resin. The prepared mixture was stirred with a speedmixer at 1200 rpm for 4 min. The epoxy resin was degassed by using vacuum for 5 min to remove the entrapped air bubbles.

#### 2.2.2 Scaffold Preparation

**Figure 1:**
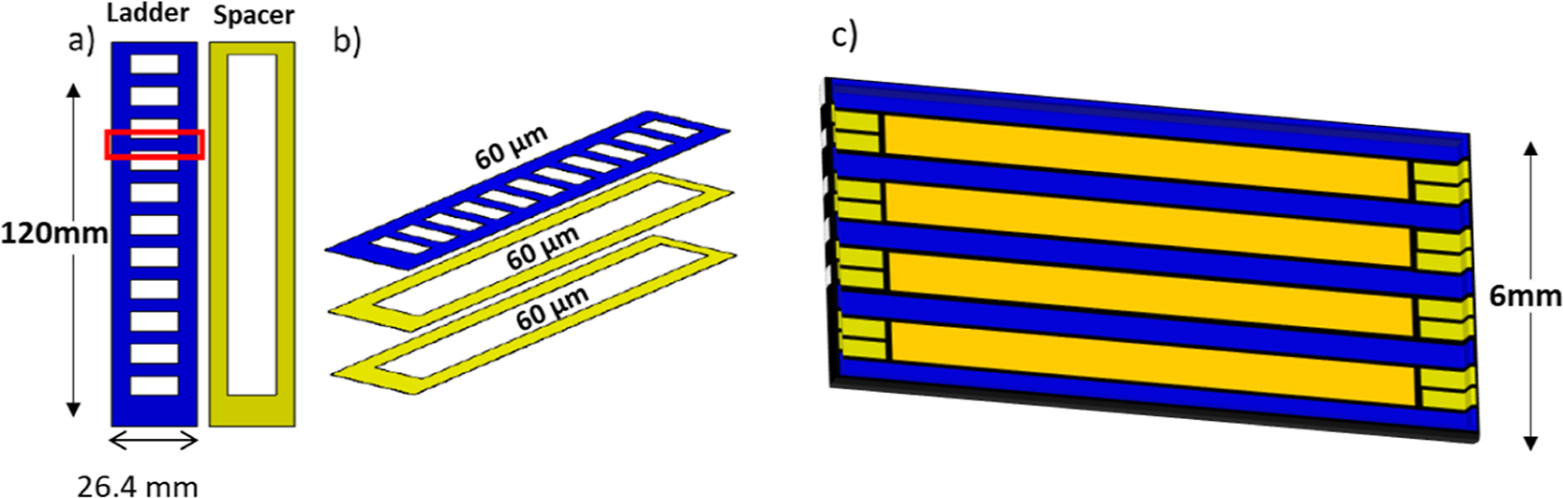
Batch manufacturing leading to 10 samples: (a) PEI film (blue) cut into “ladder–and–spacer” form of the same thickness (yellow); the red rectangle shows one sample for a single edge notched bend test; (b) stacking of the scaffold: ladder with two spacers alternating one PEI layer of same respective thickness to obtain 33 vol % PEI content and 67 vol % epoxy; and (c) cross-section of the infused scaffold (orange: infused epoxy).

PEI scaffolds were prepared by stacking the PEI films periodically in a 1:2 ratio of equal thickness as “PEI layer”, “spacer”, “spacer”, “PEI layer”, “spacer”, “spacer”, “PEI layer”, “spacer”, “spacer”, and so on (Figure 1) to obtain scaffolds with 33 vol % of PEI, respectively, with a porosity of 66% to be infiltrated by the epoxy system. After stacking, an aluminum mold was clamped and placed in an oven preheated at 220 °C for 40 min. This heating causes the thermoplastic layers to get softened and attached with each other, so that epoxy can easily flow to the monolithic polymer border. For the infiltration step, the epoxy system was placed on a heating plate and heated up to 100 °C for 2 min. It was important to heat the fresh resin upon infiltration to facilitate the resin flow and avoid air bubbles inside the resin. The epoxy system was then infused into the PEI scaffold with the help of a syringe. Infiltration occurred dominantly through capillary action. Once the infiltration step was completed, samples were placed in a preheated oven at a selected temperature to cure. The dimensions of the scaffolds were as follows: length = 120 mm, width = 26.4 mm, and thickness = 6 mm. Each scaffold gives nine samples which were used for further analysis.

#### 2.2.3 Cure Cycle

In this study, the PEI-toughened epoxy samples were cured using a two-dwell cure cycle approach already reported in the literature.^25^ The time and temperature during the curing stage (first cycle of two-dwell cure cycles, see Table 1) were varied to study their influence on the fracture toughness of the PEI-toughened epoxy system. Moreover, the temperature and time of the postcure step, also referred to as second dwell cure cycle, were selected to ultimately achieve a full cure of the epoxy/PEI system. These were all based on the cure kinetics model of the epoxy system.^25^ The details of the cure cycle are presented in Table 1. Our previous research^20^ showed that the interphase thickness varies as a function of cure temperature ranging from 120 to 200 °C. Therefore, five different (first dwell) cure temperatures of 120, 140, 160, 180, and 200 °C were selected to attain at least 70% degree of cure at the first dwell. The postcure time and temperature were selected to attain full cure of the sample.

**Table 1:** Cure Temperatures and Times Used for the Sample Preparation

***cure***		***post cure***	
*temperature*, °C	*time*, min	*temperature*, °C	*time*, min
120	95	200	40
140	75	180	30
160	75	180	20
180	45		
200	45		

#### 2.2.4 Fracture Toughness

The fracture toughness of the specimens was determined by single-edge notched bending (SENB) testing according to the ASTM D5045-99 standard.^26^ The dimensions of the SENB sample were as follows: length = 26.4 mm, width = 6 mm, and thickness = 3 mm.

**Figure 2:**
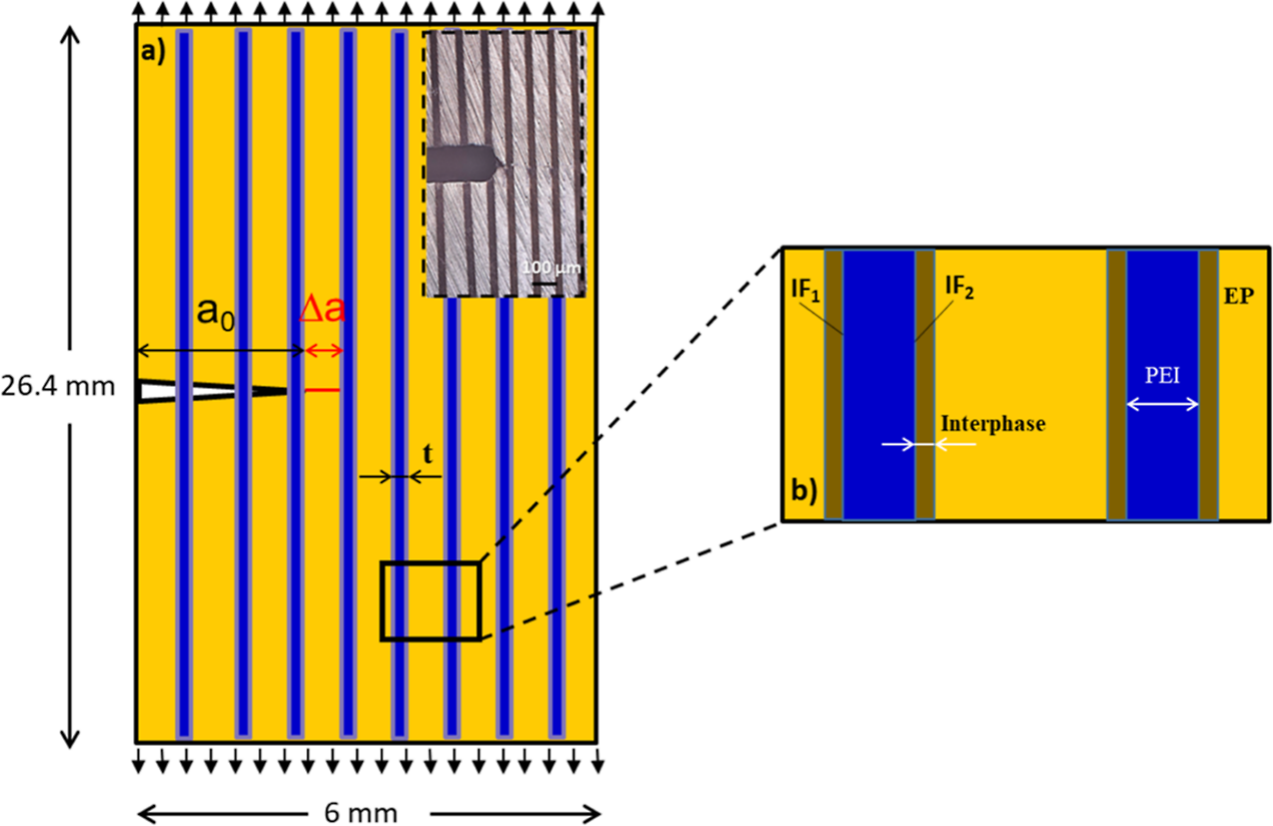
(a) Schematics of the notched sample along with a natural crack. Inset shows the notching in the real sample. (b) Schematics of different regions in the multilayered sample; PEI = poly(ether imide) (blue) EP = epoxy (yellow), IF1 = interface 1, and IF2 = interface 2.

As shown in Figure 1, each ladder scaffold was cut by a rotary cutter (machining) into 10 SENB samples. The desired thickness of each sample was ensured by manual polishing after cure. The sample was notched (*a*_0_) with the help of a saw blade of 0.12 mm thickness. Then, the notch was filled with diamond paste (DP-paste, Struers); a razor blade was inserted in the notch, and a natural crack (Δ*a*) was obtained by sliding the razor blade (see Figure 2).

The total length of the notch and natural crack (*a*_0_ + Δ*a*) was ±1.7 mm. All of the tests were conducted on a Zwick/Roell tensile test machine with a 1 kN load cell and a 10 mm/min testing speed. The device was set up according to the ASTM standard D5045-99.^26^ The resulting load–displacement curves were then translated to *K*_Ic_ and *G*_Ic_ values. The plastic zone around the crack tip in our system was estimated to be 62 μm by the Irwin model^27^ under the plane strain condition (see the Supporting Information). Therefore, linear elastic fracture mechanics (LEFM) was applied to the system because the radius of the plastic zone was smaller compared to the crack and ligament length (*r*_p_ ≪ *a*, *b*).^27^

In our study, the PEI layer thickness and cure temperature were varied. See Table 2 for an overview of the values of the parameters studied. To compare and understand the fracture toughness of the PEI-toughened epoxy scaffolds, we also produced and tested pure epoxy and pure PEI samples of the same dimensions with SENB.

**Table 2:**
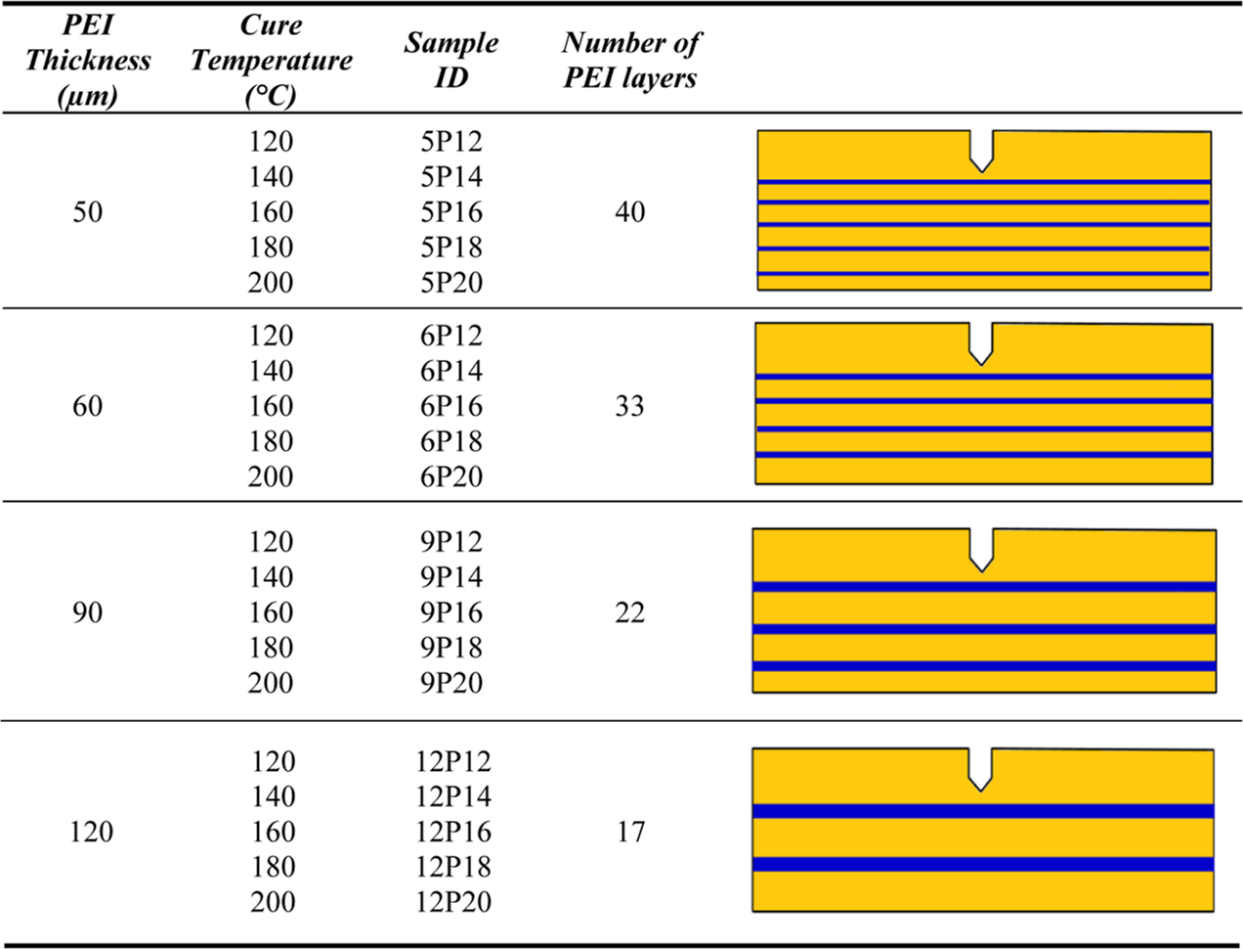
Overview of the Values of Different Parameters Used for the Experiments

#### 2.2.5 Microscopic Analysis

The interphase and morphology of the cured PEI toughened epoxy samples were studied by using field emission scanning electron microscopy (SEM) (JEOL JSM-7500F, Germany) and confocal laser scanning microscopy (CLSM) (Keyence 3D, VK-X1000). For morphological analysis, samples were embedded in a fast-cure epoxy resin, followed by grinding and polishing. *N*-Methyl-2-pyrrolidone (NMP) was used to etch the polished samples following the procedure explained elsewhere.^20^ The etched samples were coated with gold using a sputter coater and then analyzed using SEM. For fractography analysis, the cross-section of fractured samples obtained after SENB testing was also coated with gold and analyzed using SEM.

## 3 Results

### 3.1 Interphase Formation

**Figure 3:**
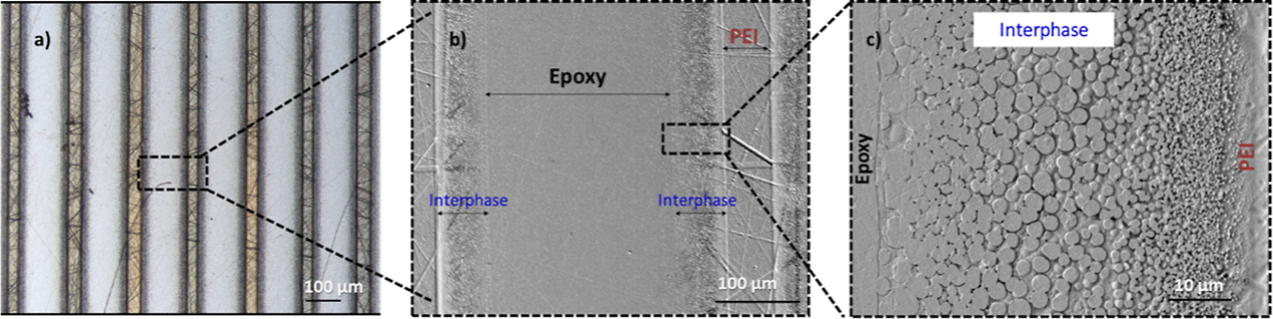
(a) CLSM and (b,c) SEM images of a PEI/epoxy system with 90 μm PEI layer cured at 160 °C.

The interphase formation between epoxy and PEI at different cure temperatures was analyzed by using CLSM and SEM. An example of the multilayer sample consisting of a PEI layer of 90 μm cured at 160 °C (1st dwell temperature) is shown in Figure 3. The CLSM image of the sample (Figure 3a) indicates the presence of parallel PEI layers evenly distributed in the brittle epoxy. At the same time, Figure 3b presents a zoomed-in SEM image of two consecutive PEI layers, along with two interphase regions, having a thickness of about 61 μm each. A distinct morphology gradient is observed in Figure 3c between a clear interface of pure epoxy (left) and pure PEI (right). This gradient morphology demonstrated the existence of epoxy-rich droplets in a PEI matrix (i.e., phase-inverted morphology) near the pure epoxy interface.^20^ The size of these epoxy droplets was observed to decrease gradually toward the pure PEI region due to the increase in PEI content. Similar results were also obtained for the samples cured at 140, 180, and 200 °C.

**Figure 4:**
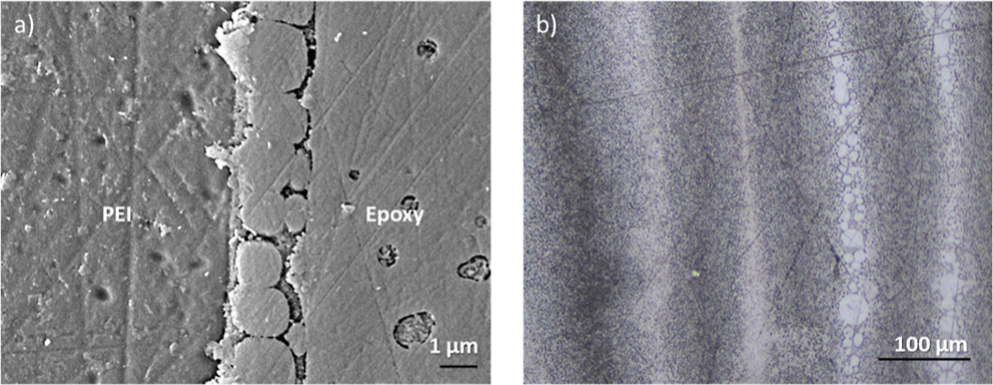
(a) SEM micrograph showing no visible gradient morphology in the interphase of a PEI/epoxy system with a 60 μm PEI layer cured at 120 °C. (b) CLSM image of a PEI/epoxy system with 50 μm PEI layer cured at 200 °C showing the complete dissolution of the PEI layers.

Most of the samples showed a distinct gradient morphology. However, two cure conditions showed different behaviors: (1) samples cured at 120 °C and (2) samples cured at 200 °C with a low PEI layer thickness. For the first case, no visible gradient morphology was observed for samples cured at 120 °C at the given resolution for all of the studied PEI layer thicknesses. For example, the SEM image of a sample with 60 μm PEI film cured at 120 °C is shown in Figure 4a. The interphase formation was observed to be significantly different, i.e., the absence of gradient morphology and a smaller interphase thickness (6.3 μm). In contrast, for the latter case for samples cured at 200 °C, the epoxy system with the lowest PEI layer thickness (50 μm) showed a complete dissolution of the PEI layer, i.e., the absence of a pure PEI region (Figure 4b).

**Figure 5:**
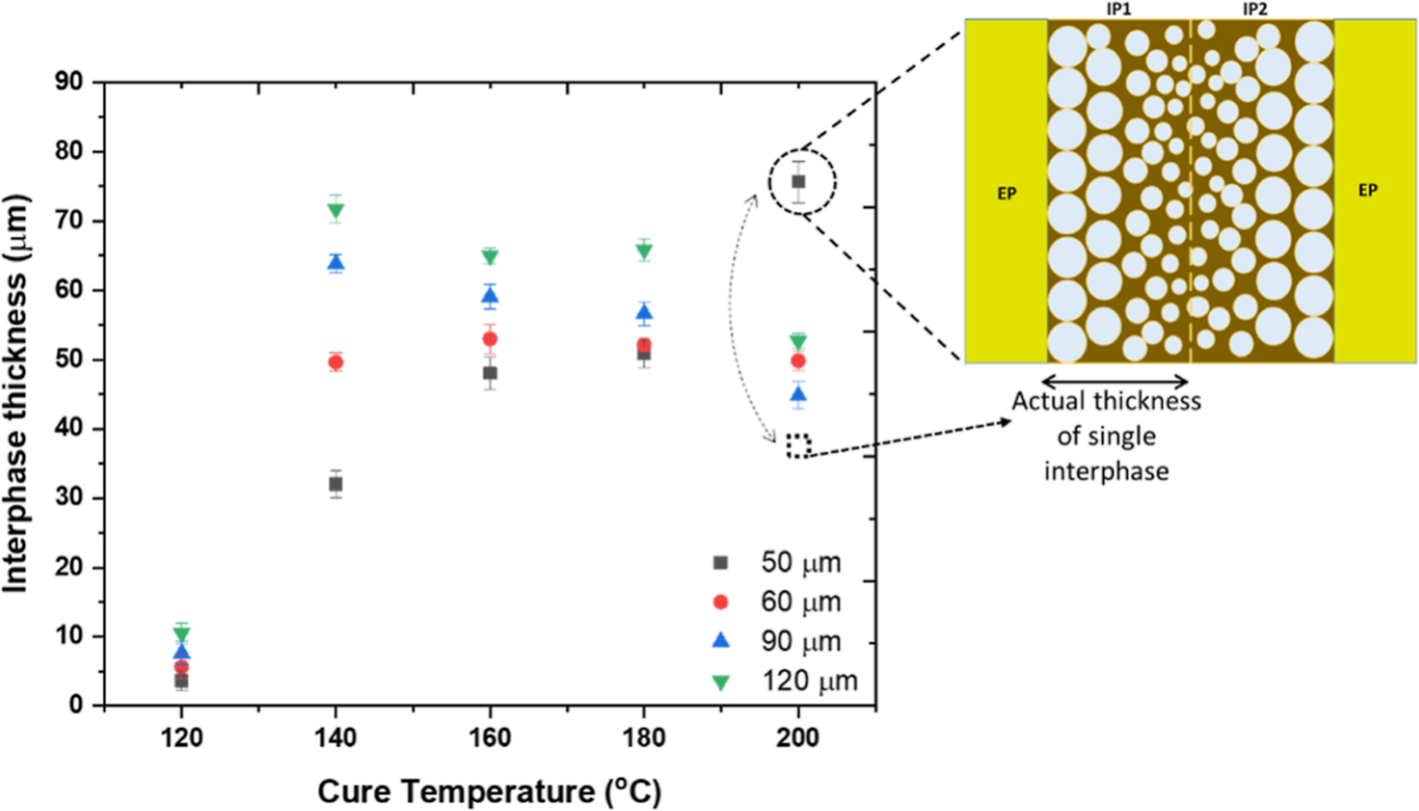
Final interphase thickness as a function of cure temperature for different PEI layer thicknesses. The schematic shows the complete dissolution of the 50 μm PEI layer and a combination of two interphases.

Figure 5 shows the interphase thickness as a function of cure temperature and the PEI layer thickness. Overall, higher PEI film thicknesses seem to result in decreased interphase dimensions with the increase in temperature. In contrast, smaller PEI film thicknesses yield competing trends in interphase thickness with cure temperatures, resulting in a maximum between 160 and 180 °C. In the case of 90 and 120 μm PEI layers, the maximum interphase thickness was observed at a cure temperature of 140 °C and decreased with higher cure temperatures. Similarly, the maximum interphase thickness was obtained at a cure temperature of 160 °C for the 60 μm PEI layer; however, there was no noticeable change in interphase thickness after further increasing the temperature to 200 °C.

On the other hand, the maximum interphase thickness for a 50 μm PEI layer seems to be found at 200 °C, which, however, was linked to the complete dissolution of the monolithic PEI layer and difficulty in analyzing the thickness of a single interphase, which would be half of the measured value. Hence, in the case of the 50 μm PEI layer, the maximum interphase thickness was evident at a cure temperature of 180 °C. This behavior shows that the critical cure temperature required for achieving maximum interphase thickness shifted toward a higher value as a function of decreasing PEI thickness. Furthermore, this graph indicates that the higher the PEI layer thickness, the higher the interphase thickness at any cure temperature. For instance, the interphase thickness is 72 μm for sample 12P14 with a PEI layer thickness of 120 μm, while for the same cure conditions, with a PEI layer thickness of 50 μm (sample 5P14), it is 33.4 μm.

### 3.2 Morphological Analysis

**Figure 6:**
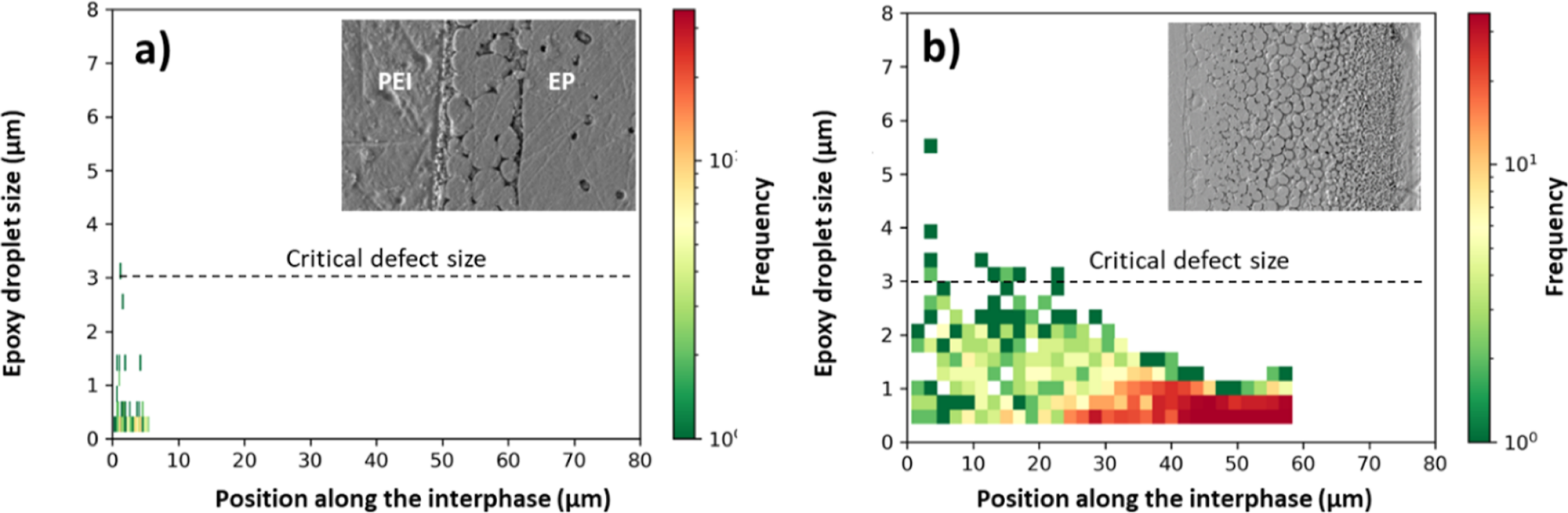
Epoxy droplet size from image analysis plotted as a function of position along the interphase (0 being pure EP) for samples with 60 μm PEI layer, cured at (a) 120 °C and (b) 180 °C. The dashed horizontal line represents the critical defect size calculated by Griffith’s theory.

The morphology gradient consists of epoxy-rich droplets in a continuous PEI-rich phase. The epoxy droplet size and frequency, calculated from blob detection with OpenCV,^20^ are plotted as a function of position along the interphase for samples cured at different temperatures (Figure 6). At 120 °C cure temperature (Figure 6a), a narrow droplet size distribution was observed due to the absence of gradient morphology, as already discussed. It is evident from Figure 6a,b that the epoxy droplet size decreased by moving toward the pure PEI phase (i.e., from left to right) for all cure temperatures. Furthermore, the droplet size and frequency of smaller droplets near the epoxy interface increased as a function of cure temperature (Figure 6b), indicating different diffusion and phase separation kinetics. The epoxy droplet size and frequency are also plotted as a function of position along the interphase for samples with a 90 μm PEI layer and 120 μm PEI layer, cured at 120 °C (Figure S4).

### 3.3 Fracture Toughness

**Figure 7:**
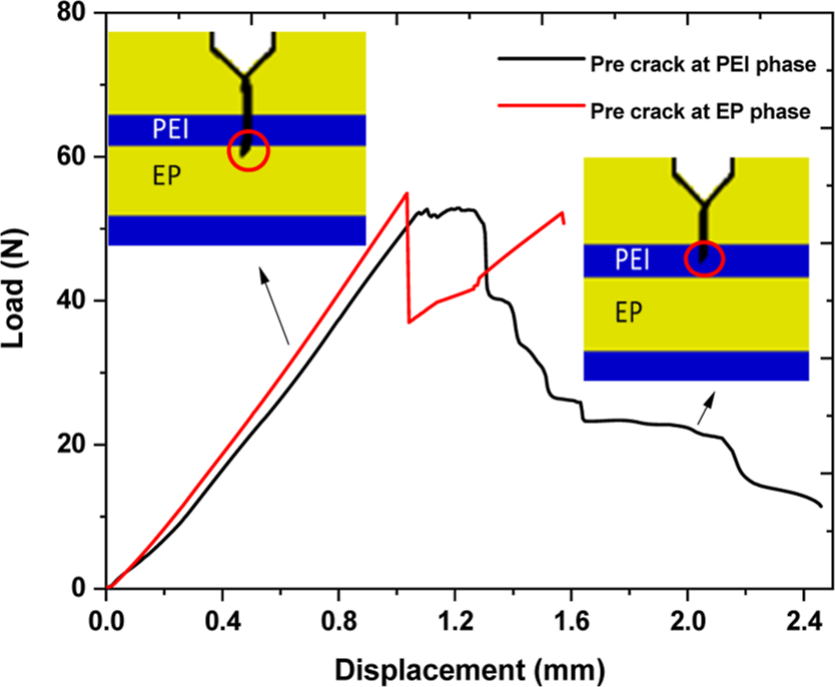
Load–displacement curve of a PEI/epoxy system with a 60 μm PEI layer cured at 140 °C has a crack tip in either the epoxy or PEI layers.

For SENB of multilayer systems, the precrack tip location in the tough PEI or brittle EP phase influences fracture behavior significantly. In Figure 7, two load–displacement curves are shown for sample 6P14, where in one sample, the crack tip is located in the epoxy layer, and in the other sample, it is located in the PEI layer. The sample having a crack tip in the epoxy layer shows a sharp decrease in load around a displacement value of 1 mm. At the same time, in the other case, the curve displays a relatively gradual decrease in load as a function of displacement, i.e., a larger area under the curve. The fracture energy in the case of a crack tip in the PEI layer is slightly higher than that in the other case. In further analysis, the load–displacement curves, which showed the presence of a crack tip in the epoxy layer, were mainly considered for all of the samples.

**Figure 8:**
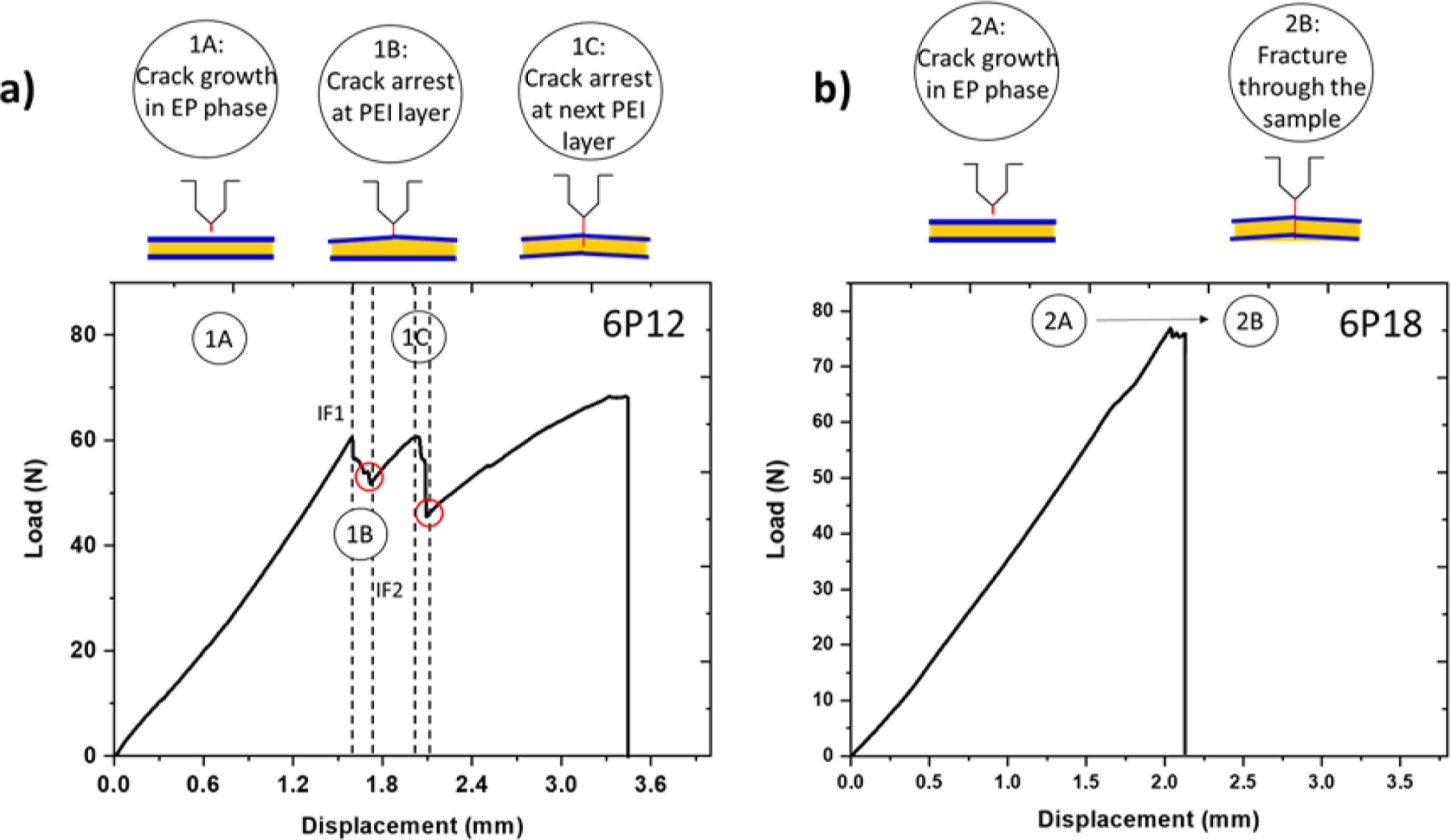
Load–displacement curve of a PEI/epoxy system with a 60 μm PEI layer (a) cured at 120 °C and (b) cured at 180 °C; the blue line represents PEI interlayers, while red circles show the crack arrest positions.

The results show a difference in the load–displacement curves for different cure temperatures. The load–displacement curves of the 6P12 and 6P18 layer architectures are compared in Figures 8a,b and S3. It is witnessed that in the case of 6P12, crack initiation happened in the epoxy phase (step 1A) as the crack tip was in the epoxy phase, which caused an increase in the load until step 1B. After that, a sharp decrease in load is found as the crack passes through interface 1 (i.e., IF1, shown in Figure 2b), and it continues to decrease until the crack tip has crossed IF2 (interface 2, see Figure 2b). This point is termed a crack arrest location and is highlighted by a red circle in Figure 8a (steps 1B and step 1C). In region 1C, a similar behavior is seen in step 1B at the start, where the crack starts to propagate again in the epoxy phase until the total fracture of the specimen. In the case of 6P18, a small area under the load–displacement curve was noticed due to the absence of crack arrest (Figure 8b). Crack initiation happened in the epoxy phase (step 2A), and the load increased until step 2B, and then, instead of a crack arrest, the crack penetrated through all interlayers.

**Figure 9:**
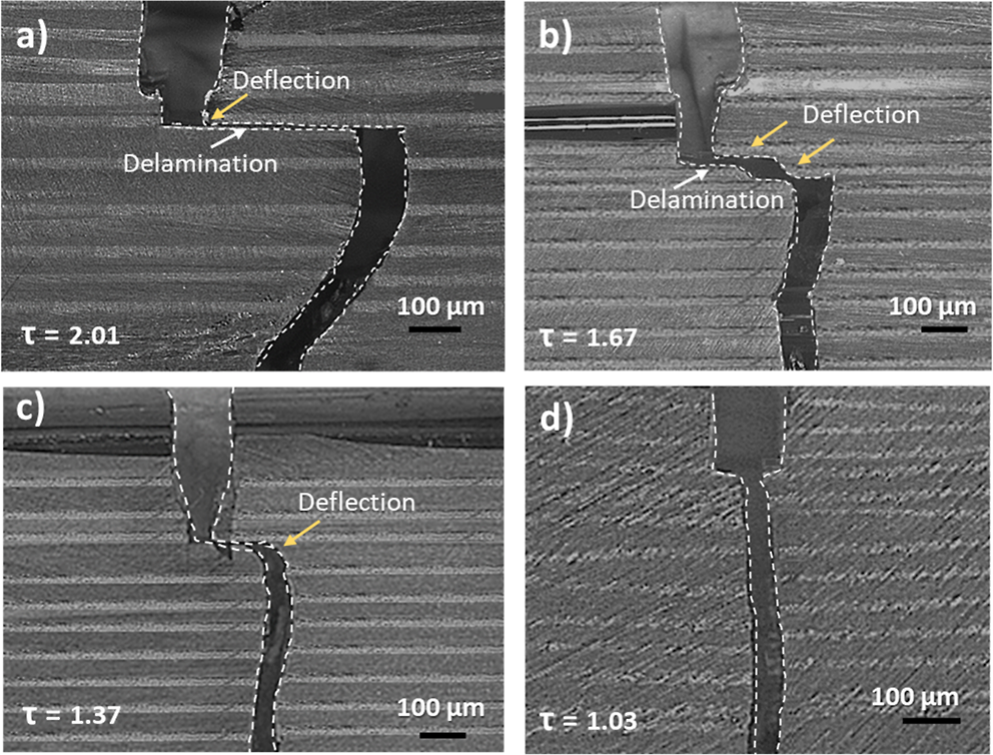
(a) CLSM images of a PEI/epoxy system with a 60 μm PEI layer cured at (a) 120 °C, (b) 140 °C, (c) 160 °C, and (d) 180 °C. Yellow arrows represent deflection, and white arrows show delamination. τ is the crack tortuosity, which is calculated by ImageJ analysis.

Figure 9 shows the microscopic images after the SENB experiment of samples cured at different cure temperatures. It is evident that several energy absorption phenomena, i.e., crack deflection and delamination, are observed in the samples. It can be seen that, at first, the crack runs straight in crack opening mode (mode I) (Figure 9a–d), tearing layer after layer of the multilayer sample until it reaches the point where the crack is deflected toward the right (yellow arrow in Figure 9a–c). Mode II fracture (shearing mode) starts taking place, causing the delamination of PEI layers (white arrows in Figure 9a,b). Hence, this behavior shows that mode I and mode II fractures are present at lower temperatures (lower interphase thickness), while mode I is dominant at a higher temperature (higher interphase thickness). In some samples, the crack continues to propagate to the point where it is deflected again (Figure 9b). It was qualitatively observed that samples display less crack deflection with the increase in the cure temperature. The crack passes through the whole sample at the highest cure temperature without any deflection, delamination, or crack arrest (Figure 9d). We further evaluated the crack tortuosity (τ), defined as the ratio of the length of the actual crack to the projected length. In the case of the lowest cure temperature, the value of crack tortuosity was 2.01, which decreased with the increase in temperature (Figure 9a–d).

**Figure 10:**
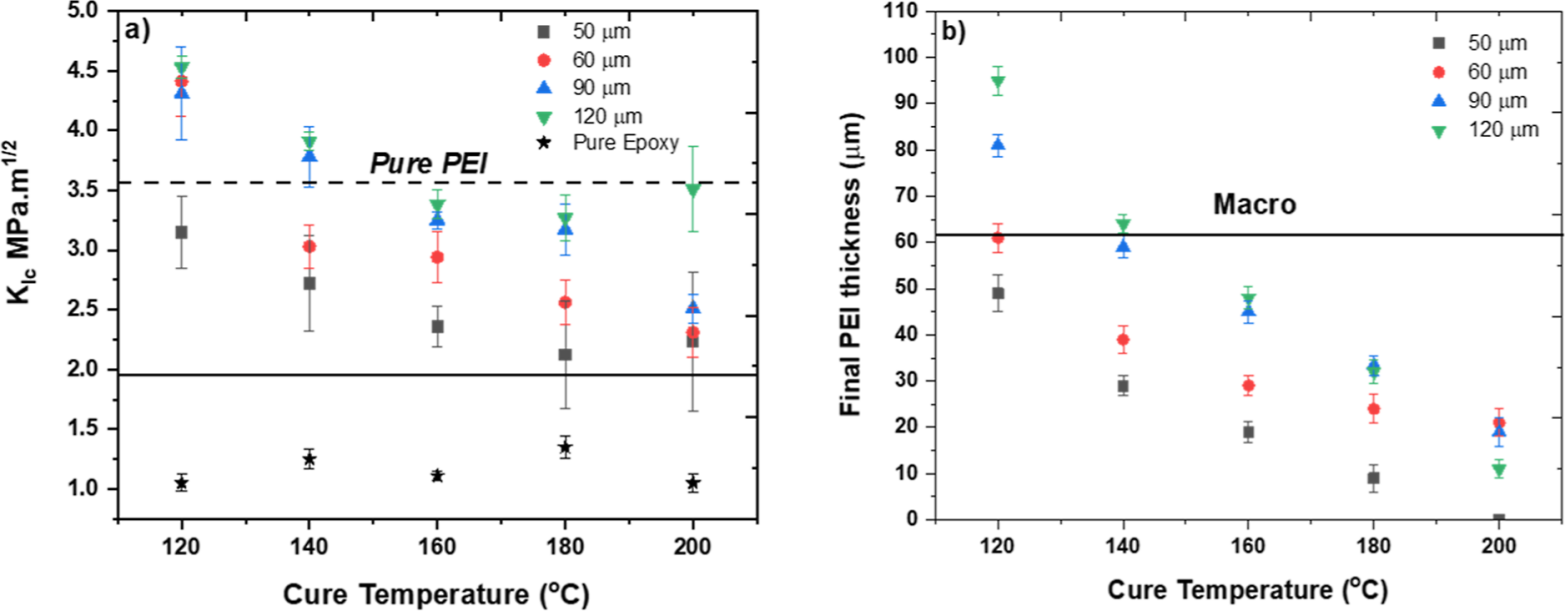
(a) Plane strain fracture toughness (*K*_Ic_) as a function of cure temperature, the dashed horizontal line represents the values of *K*_Ic_ of pure PEI, while the solid horizontal line represents *K*_Ic_ based on the rule of mixtures. (b) Remaining PEI thickness as a function of cure temperature, the solid horizontal line represents the plastic zone size of pure PEI.

The effect of cure temperature and PEI film thickness on the fracture toughness and critical energy release rate of samples is shown in Figures 10a and S1 (see the Supporting Information). The solid line in Figure 10a represents the *K*_Ic_ value calculated according to the rule of mixtures^16^
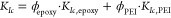
1where ϕ_epoxy_ = 2/3 and ϕ_PEI_ = 1/3 represent the volume fractions of epoxy and PEI, respectively. The rule of mixture represents the bulk resin modification, and therefore, eq 1 has been considered to compare our multilayer toughening approach with hypothetical bulk resin modification.

Both fracture toughness (*K*_Ic_) and energy release rate (*G*_Ic_) displayed a decreasing trend with the increase in cure temperature. The increase in the plane strain fracture toughness, *K*_Ic_, and energy release rate, *G*_Ic_, compared to pure PEI, particularly at the lower cure temperatures (120–140 °C), strongly suggests the synergistic benefit of multilayer architecture and morphologies present at the interphase.

The value of *K*_Ic_ and *G*_Ic_ increases with the increase in PEI layer thickness until 90 μm at a particular cure temperature, except for 120 °C. Figure 10b represents the thickness of the remaining PEI layer after curing (or after interphase formation) as a function of the cure temperature and initial PEI layer thickness. At low cure temperature (120 °C), the remaining PEI layer thickness was ≥60 μm (except for 50 μm initial PEI layer), which means the undissolved PEI thickness equal to or greater than the plastic zone size (*r*_p_ = 62 μm). At 140 °C, the remaining PEI layer thickness was similar to the plastic zone size in the case of the 90 and 120 μm initial PEI layer, while the remaining PEI layer thickness for the 50 and 60 μm initial PEI layer was less than the plastic zone. At higher cure temperatures, the remaining PEI layer thickness was well below the plastic zone size for all of the initial PEI thicknesses.

## 4 Discussion

The enhancement in fracture toughness of epoxy toughened with PEI multilayers is observed on two levels, i.e., (1) macroscale and (2) microscale.

### 4.1 Macrotoughening

The macro-scale toughening of epoxy comes from introducing tough and ductile PEI layers into the epoxy, which creates the inhomogeneity effect (i.e., mismatch in yield stress). Several studies^11−14,28^ found an increase in strain energy when a crack grows from a brittle to a ductile material, called the antishielding effect. On the other hand, the strain energy is diminished when a crack grows from the ductile to the brittle phase. This so-called shielding effect can stop the crack from growing entirely, leading to crack arrest at the interface.

It is reported that the maximum inhomogeneity effect is achieved when the thickness of the interlayer (*t*_critical_) is equal to the plastic zone size.^12,29^ At a low cure temperature (Figure 8a), multiple crack arrest positions were observed in the sample because the thickness of the remaining PEI layer was higher than the size of the plastic zone (Figure 10b). Kolednik et al. reported a loss of inhomogeneity effect due to the plastic deformation of the interlayer at a lower thickness of the soft layer.^12^ Also, in the current study, crack arrest was not achieved at higher cure temperatures (Figure 8b). This behavior was linked to the fact that the remaining PEI layer thickness was lower than the plastic zone size (Figure 10b), eventually resulting in the loss of the inhomogeneity effect and lower values of *K*_Ic_ and *G*_Ic_ (Figures 10a and S1). Therefore, this result shows that the remaining PEI thickness (affected by both initial PEI thickness and cure temperature) plays a dominant role in defining the macrotoughening phenomenon, where the critical value was found to be in the range of the plastic zone of the PEI, about ∼60 μm.

### 4.2 Microtoughening

As already discussed, liquid reactive thermoset monomers diffuse into the glassy thermoplastic and partially dissolve it. This mutual diffusion of the components creates a gradient morphology in the interfacial region (Figure S2). There are three regions of interest in this morphology: (1) PEI inclusions in the epoxy-rich phase, (2) the interphase region (with or without the gradient morphology depending upon the cure temperature), and (3) the PEI-rich phase with epoxy inclusions. The epoxy inclusions in the PEI-rich phase were not discussed in the following sections due to their negligible effect on the fracture behavior of samples. Therefore, the microscale toughening of epoxy was explained based on two remaining regions: (i) gradient morphology in the interphase region and (ii) PEI inclusions in pure epoxy.

#### 4.2.1 Interphase Region

The competition between the rate of phase separation and the curing rate governs the change in interphase thickness as a function of cure temperature (Figure 5). For instance, an increase in the interphase thickness until the cure temperatures of 140–160 °C can be attributed to the dominance of the phase separation phenomenon over the curing phenomenon. On the contrary, the curing reaction controlled the interphase thickness at higher cure temperatures (>160 °C), which resulted in smaller interphase thickness.^20^

Moreover, the interphase morphology was analyzed using SEM at different cure temperatures. The results showed the absence of a gradient morphology (i.e., only big particles) at a cure temperature of 120 °C (Figure 6a), suggesting only case II diffusion. On the other hand, a distinct gradient morphology was observed at a cure temperature of 180 °C (Figure 6b), which can be linked to the combination of case I and case II diffusion.^20^ A similar gradient morphology was also reported in the literature for epoxy/PSU and epoxy/PEI systems.^7,20^ SEM analysis also revealed the formation of a higher number of smaller droplets at higher cure temperatures (Figure 6b), which can be associated with the hindered mobility of polymeric chains and reduced growth of particles after the phase separation phenomenon.^30^ The first interesting observation was that the epoxy system modified with the lowest PEI layer thickness (50 μm) and cured at 200 °C showed a complete dissolution of the PEI layer (Figure 4b). In this case, the *K*_Ic_ value is similar to the value reported in the literature for bulk modification of the trifunctional epoxy resin using PEI in the range of 30 to 35 vol %.^31^ Moreover, this *K*_Ic_ value is similar to the value calculated by the ideal mixture rule (see Figure 10a), which shows that the discussed macrotoughening phenomena are insignificant in this case.

**Figure 11:**
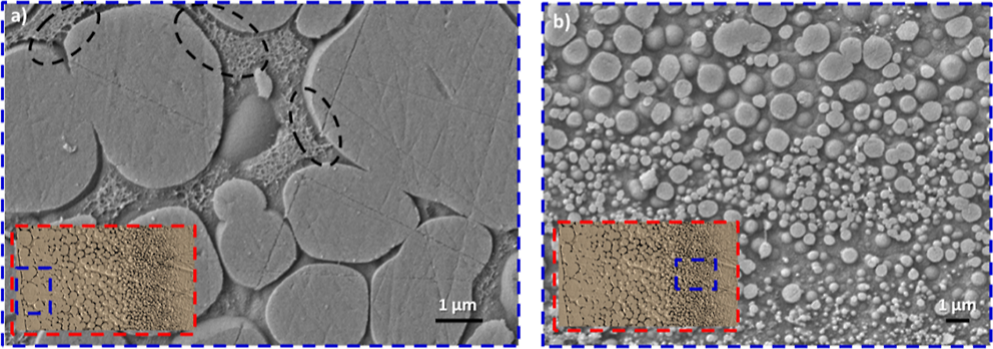
SEM micrograph showing a region within the interphase of a PEI/epoxy system obtained at 160 °C: (a) epoxy particles near the pure epoxy layer and (b) epoxy particles near the pure PEI region. Dotted circles show the presence of a gap around epoxy droplets, which was a pure PEI region washed away during NMP treatment.

The size of the epoxy particles in the gradient interphase governs their connectivity with the thermoplastic phase. Figure 11a shows that big particles (≥3 μm) formed near the epoxy layer are fully surrounded by pure PEI, hence showing more connectivity to the thermoplastic phase. The gap around the epoxy particles (circles with dotted lines) represents the pure PEI phase which was washed away during polishing with NMP. In contrast, small epoxy particles, ≤3 μm (present near the PEI layer), show no connectivity with the thermoplastic phase (Figure 11b).

As shown in Figure 6a, at a low cure temperature, the interphase region only consists of bigger particles, which result in enhanced connectivity with the thermoplastic phase and may result in a stronger interface. On the contrary, at higher cure temperatures, less connectivity was observed due to the higher frequency of smaller particles than bigger particles (Figure 6b), which can lead to a weaker interface. The strong interface between big epoxy particles and PEI formed at lower cure temperatures showed enhanced crack growth resistance due to the different energy-dissipating mechanisms, such as crack deflection and delamination.^32^ A crack generally propagates in the direction of the least resistance. Therefore, at lower temperatures, the crack prefers to propagate around the interface instead of propagating through the interface due to the weak adhesion (Figure 9a,b), which results in a larger distance covered by the crack (i.e., higher crack tortuosity). The interface becomes weaker with further increase of the temperature, and these energy dissipation mechanisms become less prominent (Figure 9c). At the highest cure temperature (Figure 9d), the crack easily propagates through the interface due to small epoxy particles in an interphase region, which eventually provides the least resistance toward crack growth.

**Figure 12:**
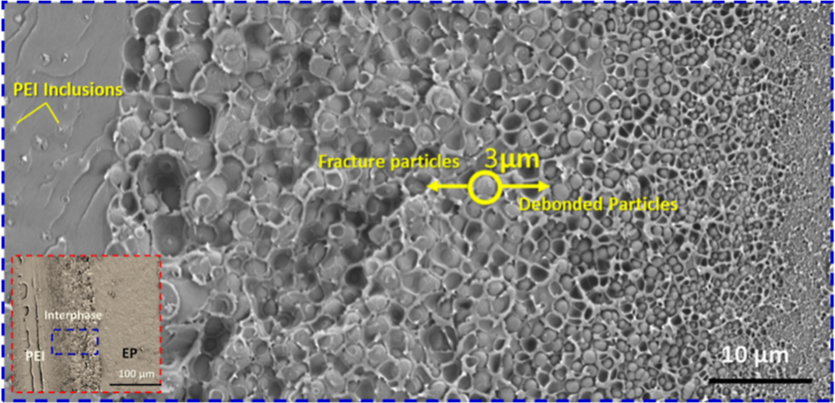
SEM micrograph of the PEI/epoxy system with 90 μm PEI layer cured at 160 °C. The yellow circle represents the critical defect size of epoxy particles estimated by Griffith’s theory (see the Supporting Information).

Also, fractography of the fracture surfaces helps us better understand the underlying reasons for the observed differences at the microscale. The gradient interphase shows different morphologies throughout its thickness, depending on the concentration of epoxy and PEI. SEM micrograph of the multilayer fracture surface of sample 9P16 is shown in Figure 12. It shows that a gradient phase inverted morphology of epoxy particles is present in the interphase region.

Observing the fracture surface, the interphase region in the vicinity of the epoxy layer demonstrates plastic deformation of the ductile PEI phase and subsequent fracture of the big epoxy particles. On the other hand, debonding of smaller epoxy particles is evident near the pure PEI layer because the size of the epoxy particles was smaller than the critical defect size (∼3 μm) calculated by Griffith’s theory.^33^ At low cure temperatures (for instance, 120 °C), the interphase typically shows the presence of only bigger particles (see Figure 4a), which means the fracture of epoxy particles is the primary fracture mechanism since their size was larger than the critical defect size (∼3 μm).

#### 4.2.2 PEI Inclusions in Pure Epoxy

**Figure 13:**
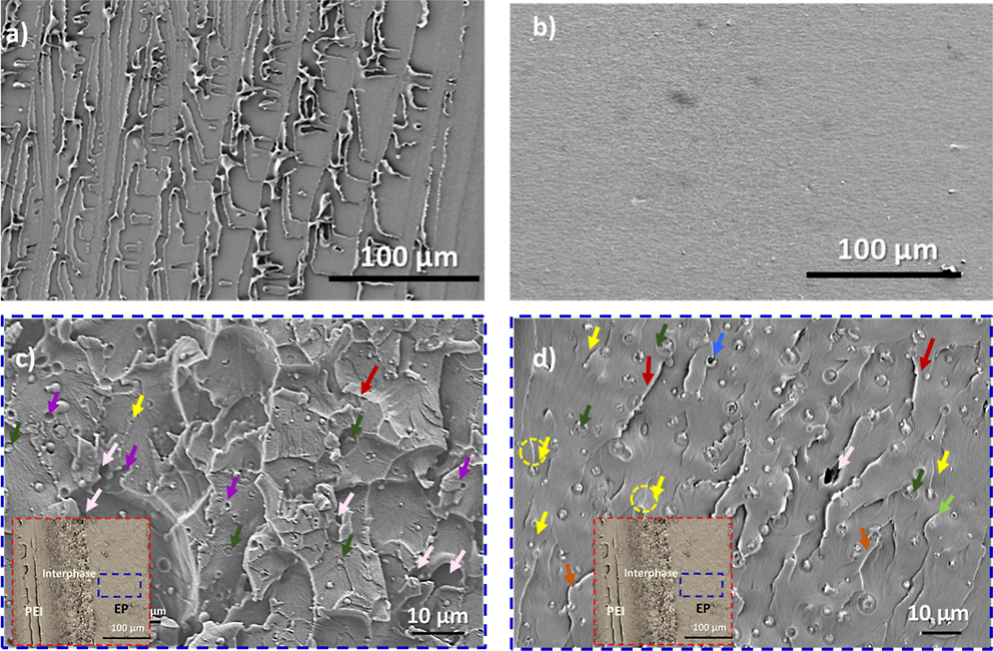
(a) SEM micrograph of pure PEI, (b) SEM micrograph of pure epoxy, and (c) SEM micrograph of the epoxy-rich phase with PEI inclusions having a 60 μm PEI film cured at (c) 140 °C and (d) 180 °C. Purple arrows show debonding of particles, dark green arrows show the fracture of particles, red arrows show shear banding, orange arrows show crack bridging, light green arrows show crack deflection, light pink arrows show cavitation, and yellow arrows show crack pinning.

Figure 13a shows the pure thermoplastic (PEI) fracture surface. Figure 13b shows the untoughened epoxy resin samples, demonstrating a relatively flat and featureless fracture surface, i.e., a typical characteristic of brittle fracture.^16,34^ The fracture surfaces of the epoxy-rich phase with PEI inclusions show different toughening mechanisms at different cure temperatures. The epoxy-rich phase shows a ductile deformation (3D pattern) in the case of low cure temperature (Figure 13c). Moreover, most of the PEI particles in the epoxy phase are debonded (identified by the purple arrows in Figure 13c), while some are fractured (dark green arrows in Figure 13c). Stress triaxiality is strongly reduced during crack propagation due to the particle debonding and fracture under plane strain conditions. As a result, nonhomogeneous stress states cause localized regions of plastic deformation in the epoxy matrix known as shear bands.^23,35^ The wrinkled texture of the fracture surfaces indicates shear bands (red arrows in Figure 13c,d) leading to ductile behavior.

On the contrary, the fracture surface at the higher cure temperature shows a more river-like pattern with fractured PEI particles due to plastic deformation (Figure 13d). A limited number of particle cavitations are visible at high cure temperatures, which confirms that in these samples, the bridging of PEI particles (orange arrows in Figure 13d) is one of the most dominant energy dissipation mechanisms in the event of a crack. Other fracture mechanisms, including crack deflection (light green arrows in Figure 13d), cavitation (light pink arrows in Figure 13c,d), and crack pinning (yellow arrows in Figure 13c,d), are also evident. In the case of crack deflection, the crack is diverted by the PEI particle, increasing the crack surface area. Crack pinning is indicated by the presence of characteristic tails (yellow circles in Figure 13d).

### 4.3 Effectiveness of Micro vs Macro Toughening

**Figure 14:**
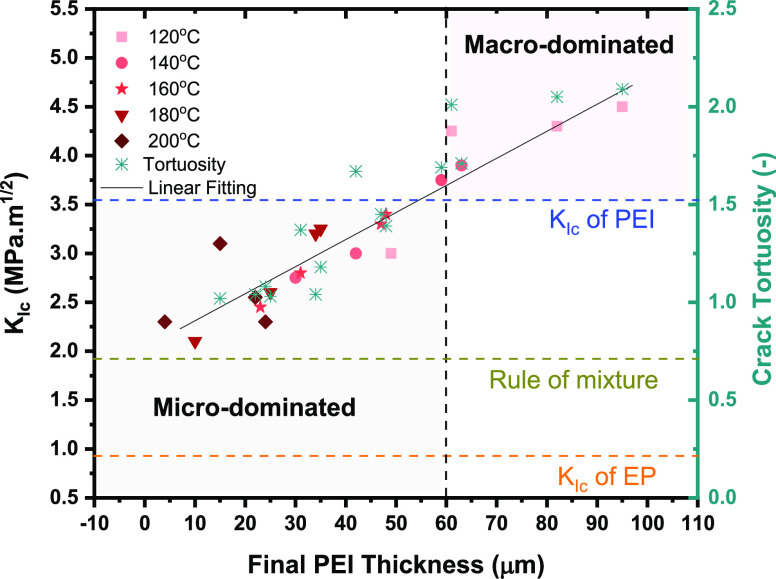
Fracture toughness (*K*_Ic_) and tortuosity as a function of the final PEI thickness obtained after curing. The vertical dashed line represents the size of the plastic zone (*r*_p_ = 62 μm) of PEI; the horizontal blue and orange lines represent the fracture toughness of pure PEI and pure epoxy, respectively. The solid black line represents the linear fitting. The green dash line represents the theoretical values from the rule of mixtures.

To identify the main controlling parameter (cure temperature or PEI film thickness) for modifying the fracture toughness of epoxy, plane strain fracture toughness, *K*_Ic_, is plotted as a function of the final PEI thickness after curing (see Figure 14). This graph shows that the fracture toughness is mainly controlled by the final PEI thickness, i.e., higher fracture toughness at higher final PEI film thickness. However, this final PEI film thickness can either be varied by changing the initial PEI film thickness or by the cure temperature. The higher plane strain fracture toughness, *K*_Ic_, and energy release rate, *G*_Ic_, compared to pure PEI, particularly at the higher final PEI film thickness (larger than 62 μm), strongly suggests the dominance of the macrotoughening phenomenon coming from multilayer architecture (Figure 14). This dominance mainly comes from the effective inhomogeneity effect due to the critical remaining PEI layer thickness.

On the other hand, when the final PEI thickness is smaller than 62 μm, the fracture toughness of modified epoxy is lower than pure PEI but still higher than pure epoxy and “bulk toughened” systems with the same volume percentage, which shows that the governing mechanism, in this case, is microtoughening. This mechanism mainly comes from the presence of bigger epoxy particles in gradient morphology, which plays a significant role during fracture (i.e., fracture of particles) and gives higher crack resistance. Moreover, with a decrease in the final PEI thickness, the spacing between the two PEI layers increases, eventually resulting in lower fracture toughness. Likewise, Kolednik et al. and several other studies found that the lower the spacing between two interlayers, the more the fracture toughness for the given thickness.^10,12,36^ Furthermore, by decreasing the final PEI film thickness, the mechanical properties of PEI-toughened epoxy approach the theoretical values of *K*_Ic_ and *G*_Ic_ (Figure 14) obtained from the rule of mixtures (eq 1). In literature,^16^ the maximum *K*_Ic_ value of epoxy, achieved so far, by using PEI as a bulk modifier is 3.1 MPa m^1/2^, while in this study, the maximum reported fracture toughness value is 4.54 MPa m^1/2^ (47% higher).

**Figure 15:**
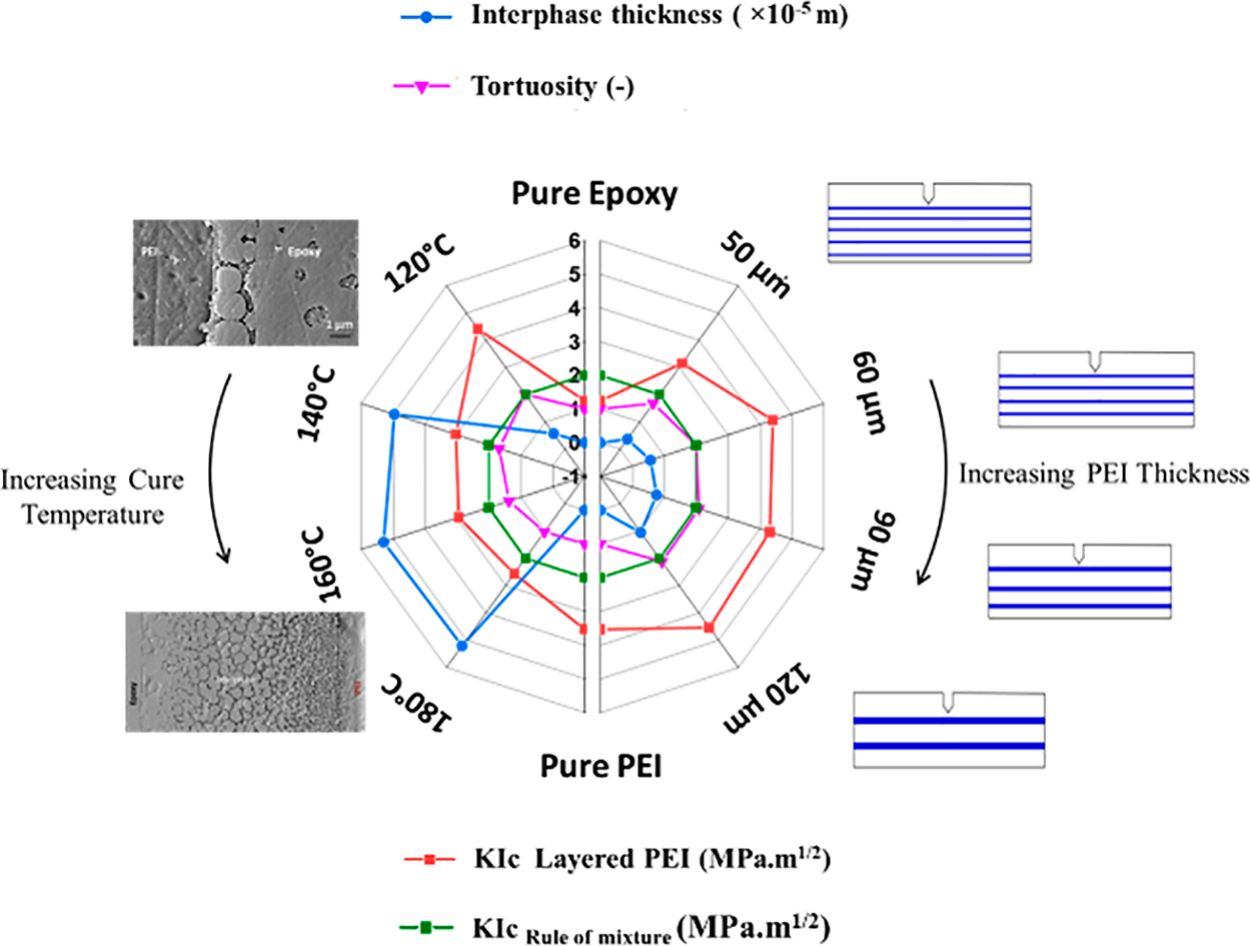
Fracture toughness (*K*_Ic_), *K*_Ic_ obtained by the rule of mixture, interphase thickness, and tortuosity as a function of increasing PEI film thickness at a fixed cure temperature of 120 °C (right side) and as a function of increasing cure temperature at a fixed PEI film thickness of 60 μm (left side).

Figure 15 presents a summary of mechanical properties (*K*_Ic_) as a function of processing parameters (cure temperature), morphology (interphase thickness), and architectural configuration (thickness of the PEI film). This graph shows that a lower interphase thickness results in better fracture toughness of the epoxy system (Figure 15) because at a higher interphase thickness, the frequency of smaller particles dominates the bigger particles and gives poor crack resistance. This is also seen from the tortuosity values, where higher values indicate higher fracture toughness. This study shows that the final PEI film thickness (controlled by cure temperature and initial PEI film thickness) significantly affects the mechanical properties of epoxy.

## 5 Conclusions

Epoxies with high cross-linking densities are brittle and typically have a low fracture toughness. Numerous approaches have been studied to increase epoxy’s fracture toughness, such as the inclusion of a secondary phase within the epoxy resin through bulk resin modification. In contrast to bulk modification, hierarchically toughened structures were also used to toughen the epoxy resin. This study comprises an understanding of the effect of macro- and microtoughening on the fracture toughness of an epoxy system by using thermoplastic PEI multilayers. The fracture toughness of the modified epoxy system was investigated as a function of varying cure temperature (120–200 °C) and PEI film thickness (50–120 μm). PEI scaffolds were prepared by stacking the PEI films periodically in a 1:2 ratio of equal thickness, which resulted in scaffolds with 33 vol % of PEI and 66 vol % of epoxy. This study cured the PEI-toughened epoxy samples using a two-dwell cure cycle.

The result shows that the fracture toughness was mainly controlled by the final PEI thickness, i.e., higher fracture toughness (4.53 MPa m^1/2^) at higher final PEI film thickness (95 μm). However, changing the initial PEI film thickness or cure temperature can control this final PEI film thickness. Compared to pure PEI, the higher fracture toughness of toughened epoxy, particularly at the higher final PEI film thickness (larger than 62 μm), strongly suggested the occurrence of both macro- and microtoughening. However, the dominant mechanism is macrotoughening coming from the multilayer architecture. This dominance mainly comes from the effective inhomogeneity effect due to the critical final PEI layer thickness. On the other hand, the fracture toughness of modified epoxy was lower than pure PEI but still higher than pure epoxy and bulk-modified with a similar volume fraction when the final PEI thickness was smaller than 62 μm, which showed that the only governing mechanism was microtoughening. This mechanism mainly comes from bigger epoxy particles in gradient morphology, which plays a significant role during fracture (i.e., fracture of particles) and gives higher crack resistance. This results show that for high-temperature processing, the initial PEI film thickness has to be selected such that the remaining film thickness allows us to obtain the synergy between micro- and macrotoughening.

Moreover, with the decrease in the final PEI thickness, the spacing between the two PEI layers increases, eventually resulting in lower fracture toughness. However, in the current study, the influence of interlayer spacing cannot be investigated because of the fixed EP/PEI volume ratio. Therefore, in the future, to effectively understand the influence of interlayer spacing on the fracture toughness of epoxy, further research needs to be performed without fixing the volumetric ratio between epoxy and PEI. Furthermore, by decreasing the final PEI film thickness, the mechanical properties of PEI-toughened epoxy approach the theoretical values of *K*_Ic_ and *G*_Ic_ obtained from the mixture rule. This study provides an understanding of the hierarchical toughening of epoxy using a multilayer system which can further be explored in the field of fiber-reinforced composites for aerospace applications.

## Supporting Information Available

Energy release rate (*G*_Ic_) as a function of cure temperature for different PEI layer thicknesses, SEM micrograph showing phase-inverted morphology (epoxy-rich particles densely dispersed in a continuous PEI-rich matrix) of a PEI-epoxy interphase obtained at 180 °C cure temperature, load–displacement curves of three samples of the PEI/epoxy system with a 60 μm PEI layer cured at 120 and at 180 °C, epoxy droplet size from image analysis plotted as a function of position along the interphase (0 being pure EP) for samples with a 90 μm PEI layer and 120 μm PEI layer cured at 120 °C, and calculation of plastic zone and critical defect size (PDF)

## References

[ref1] Kim J.; Baillie C.; Poh J.; Mai Y.-W. Fracture Toughness of CFRP with Modified Epoxy Resin Matrices. Compos. Sci. Technol. 1992, 43 (3), 283–297. 10.1016/0266-3538(92)90099-O.

[ref2] Chen Y.; Zhang H.-B.; Yang Y.; Wang M.; Cao A.; Yu Z.-Z. High-Performance Epoxy Nanocomposites Reinforced with Three-Dimensional Carbon Nanotube Sponge for Electromagnetic Interference Shielding. Adv. Funct. Mater. 2016, 26 (3), 447–455. 10.1002/adfm.201503782.

[ref3] Farooq U.; Teuwen J.; Dransfeld C. Toughening of Epoxy Systems with Interpenetrating Polymer Network (IPN): A Review. Polymers 2020, 12 (9), 1908 10.3390/polym12091908. 32847125 PMC7564612

[ref4] Wu S.; Guo Q.; Kraska M.; Stühn B.; Mai Y.-W. Toughening Epoxy Thermosets with Block Ionomers: The Role of Phase Domain Size. Macromolecules 2013, 46 (20), 8190–8202. 10.1021/ma401478t.

[ref5] Frigione M.; Mascia L.; Acierno D. Oligomeric and Polymeric Modifiers for Toughening of Epoxy Resins. Eur. Polym. J. 1995, 31 (11), 1021–1029. 10.1016/0014-3057(95)00091-7.

[ref6] Van Velthem P.; Ballout W.; Daoust D.; Sclavons M.; Cordenier F.; Henry E.; Dumont D.; Destoop V.; Pardoen T.; Bailly C. Influence of Thermoplastic Diffusion on Morphology Gradient and on Delamination Toughness of RTM-Manufactured Composites. Composites, Part A 2015, 72, 175–183. 10.1016/j.compositesa.2015.02.012.

[ref7] Vandi L.-J.; Hou M.; Veidt M.; Truss R.; Heitzmann M.; Paton R. Interface Diffusion and Morphology of Aerospace Grade Epoxy Co-Cured with Thermoplastic Polymers. In 28th International Congress of the Aeronautical Sciences (ICAS); Brisbane, Australia, 2012; pp 23–28.

[ref8] Wang J.; Pozegic T. R.; Xu Z.; Nigmatullin R.; Harniman R. L.; Eichhorn S. J. Cellulose Nanocrystal-Polyetherimide Hybrid Nanofibrous Interleaves for Enhanced Interlaminar Fracture Toughness of Carbon Fibre/Epoxy Composites. Compos. Sci. Technol. 2019, 182, 107744 10.1016/j.compscitech.2019.107744.

[ref9] Grossman M.; Pivovarov D.; Bouville F.; Dransfeld C.; Masania K.; Studart A. R. Hierarchical Toughening of Nacre-Like Composites. Adv. Funct. Mater. 2019, 29 (9), 1806800 10.1002/adfm.201806800.

[ref10] Kolednik O.; Kasberger R.; Sistaninia M.; Predan J.; Kegl M. Development of Damage-Tolerant and Fracture-Resistant Materials by Utilizing the Material Inhomogeneity Effect. J. Appl. Mech. 2019, 86 (11), 111004 10.1115/1.4043829.

[ref11] Wiener J.; Arbeiter F.; Kolednik O.; Pinter G. Influence of Layer Architecture on Fracture Toughness and Specimen Stiffness in Polymer Multilayer Composites. Mater. Des. 2022, 219, 110828 10.1016/j.matdes.2022.110828.

[ref12] Sistaninia M.; Kasberger R.; Kolednik O. To the Design of Highly Fracture-Resistant Composites by The Application of The Yield Stress Inhomogeneity Effect. Compos. Struct. 2018, 185, 113–122. 10.1016/j.compstruct.2017.10.081.

[ref13] Zechner J.; Kolednik O. Fracture Resistance Of Aluminum Multilayer Composites. Eng. Fract. Mech. 2013, 110, 489–500. 10.1016/j.engfracmech.2012.11.007.

[ref14] Fratzl P.; Gupta H. S.; Fischer F. D.; Kolednik O. Hindered Crack Propagation in Materials with Periodically Varying Young’s Modulus—Lessons From Biological Materials. Adv. Mater. 2007, 19 (18), 2657–2661. 10.1002/adma.200602394.

[ref15] Ševeček O.; Kotoul M.; Leguillon D.; Martin E.; Bermejo R. Assessment of Crack-Related Problems in Layered Ceramics using the Finite Fracture Mechanics and Coupled Stress-Energy Criterion. Procedia Struct. Integr. 2016, 2, 2014–2021. 10.1016/j.prostr.2016.06.253.

[ref16] Harismendy I.; Del Rio M.; Marieta C.; Gavalda J.; Mondragon I. Dicyanate Ester-Polyetherimide Semi-Interpenetrating Polymer Networks. II. Effects of Morphology on the Fracture Toughness and Mechanical Properties. J. Appl. Polym. Sci. 2001, 80 (14), 2759–2767. 10.1002/app.1391.

[ref17] Harismendy I.; Del Rio M.; Eceiza A.; Gavalda J.; Gomez C.; Mondragon I. Morphology and Thermal Behavior of Dicyanate Ester-Polyetherimide Semi-IPNS Cured at Different Conditions. J. Appl. Polym. Sci. 2000, 76 (7), 1037–1047. 10.1002/(SICI)1097-4628(20000516)76:7<1037::AID-APP7>3.0.CO;2-Y.

[ref18] Heitzmann M. T.; Hou M.; Veidt M.; Vandi L. J.; Paton R. Morphology of an Interface between Polyetherimide and Epoxy Prepreg. Adv. Mater. Res. 2011, 393–395, 184–188. 10.4028/www.scientific.net/AMR.393-395.184.

[ref19] Tao Q.; Wang M.; Gan W.; Yu Y.; Tang X.; Li S.; Zhuang J. Studies on the Phase Separation of Poly (Ether Imide)-Modified Cyanate Ester Resin. J. Macromol. Sci., Part A: Pure Appl.Chem. 2003, 40 (11), 1199–1211. 10.1081/MA-120024834.

[ref20] Farooq U.; Heuer S. N.; Teuwen J.; Dransfeld C. Effect of a Dwell Stage in the Cure Cycle on the Interphase Formation in a Poly (Ether Imide)/High T G Epoxy System. ACS Appl. Polym. Mater. 2021, 3 (12), 6111–6119. 10.1021/acsapm.1c00956.

[ref21] Daelemans L.; Van Paepegem W.; D’hooge D. R.; De Clerck K. Excellent Nanofiber Adhesion for Hybrid Polymer Materials with High Toughness based on Matrix Interdiffusion during Chemical Conversion. Adv. Funct. Mater. 2019, 29 (8), 1807434 10.1002/adfm.201807434.

[ref22] Brauner C.; Nakouzi S.; Zweifel L.; Tresch J. Co-Curing Behaviour of Thermoset Composites with a Thermoplastic Boundary Layer for Welding Purposes. Adv. Compos. Lett. 2020, 29, 2633366X2090277 10.1177/2633366x20902777.

[ref23] Zotti A.; Zuppolini S.; Zarrelli M.; Borriello A. Fracture Toughening Mechanisms in Epoxy Adhesives. Adhesives-Applications and Properties; IntechOpen, 2016; Vol. 1, p 257.

[ref24] Bray D.; Dittanet P.; Guild F.; Kinloch A.; Masania K.; Pearson R.; Taylor A. The Modelling of the Toughening of Epoxy Polymers via Silica Nanoparticles: The Effects of Volume Fraction and Particle Size. Polymer 2013, 54 (26), 7022–7032. 10.1016/j.polymer.2013.10.034.

[ref25] Teuwen J.; Asquier J.; Inderkum P.; Masania K.; Brauner C.; Villegas I.; Dransfeld C. Gradient Interphases Between High-Tg Epoxy and Polyetherimide For Advanced Joining Processes. In ECCM18: 18th European Conference on Composite Materials; 2018.

[ref26] ASTM. Standard Test Methods for Plane-Strain Fracture Toughness and Strain Energy Release Rate of Plastic Materials; ASTM D5045-99, 2007.

[ref27] Kolednik O. Fracture Mechanics. In Wiley Encyclopedia Of Composites; Nicolais L., Ed., Wiley, 2012; pp 1–16.

[ref28] Kopp J.-B.; Girardot J. Dynamic Fracture In a Semicrystalline Biobased Polymer: An Analysis of the Fracture Surface. Int. J. Fract. 2020, 226 (1), 121–132. 10.1007/s10704-020-00482-y.

[ref29] Sistaninia M.; Kolednik O. Effect of a Single Soft Interlayer on the Crack Driving Force. Eng. Fract. Mech. 2014, 130, 21–41. 10.1016/j.engfracmech.2014.02.026.

[ref30] Surendran A.; Joy J.; Parameswaranpillai J.; Anas S.; Thomas S. An Overview of Viscoelastic Phase Separation in Epoxy Based Blends. Soft Matter 2020, 16 (14), 3363–3377. 10.1039/C9SM02361E. 32215406

[ref31] Hourston D.; Lane J. The Toughening of Epoxy Resins with Thermoplastics: 1. Trifunctional Epoxy Resin-Polyetherimide Blends. Polymer 1992, 33 (7), 1379–1383. 10.1016/0032-3861(92)90110-I.

[ref32] Waly C.; Petersmann S.; Arbeiter F. Multimaterial Extrusion-Based Additive Manufacturing of Compliant Crack Arrester: Influence of Interlayer Length, Thickness, and Applied Strain Rate. Adv. Eng. Mater. 2022, 25, 2101703 10.1002/adem.202101703.

[ref33] Zehnder A. T. Griffith Theory of Fracture. In Encyclopedia Of Tribology; Wang Q. J., Chung Y.-W., Eds.; Springer US: Boston, MA, 2013; pp 1570–1573.

[ref34] Ma H.; Aravand M. A.; Falzon B. G. Phase Morphology and Mechanical Properties of Polyetherimide Modified Epoxy Resins: A Comparative Study. Polymer 2019, 179, 121640 10.1016/j.polymer.2019.121640.

[ref35] Wang C.; Sun Q.; Lei K.; Chen C.; Yao L.; Peng Z. Effect of Toughening with Different Liquid Rubber on Dielectric Relaxation Properties of Epoxy Resin. Polymers 2020, 12 (2), 433 10.3390/polym12020433. 32059507 PMC7077633

[ref36] Kolednik O.; Predan J.; Fischer F. D.; Fratzl P. Improvements of Strength and Fracture Resistance by Spatial Material Property Variations. Acta Mater. 2014, 68, 279–294. 10.1016/j.actamat.2014.01.034.

