# Supplementary material for: Synergistic Toughening of Epoxy through Layered Poly(ether imide) with Dual-Scale Morphologies
Source: ACS Appl Mater Interfaces. 2023 Nov 2;15(45):53074–85. doi: 10.1021/acsami.3c10096 (PMC10658453; doi:10.1021/acsami.3c10096)
Supplement: Supplementary file 1 — am3c10096_si_001.pdf [file am3c10096_si_001.pdf]

Supporting information

Synergistic toughening of epoxy through layered polyetherimide with dual-scale morphologies

Ujala Farooq,[1*] Ekaterina Sakarinen,[2] Julie Teuwen,[1] René Alderliesten,[1] Clemens Dransfeld[1*]

1 Faculty of Aerospace Engineering, Aerospace Manufacturing Technologies, Delft University of Technology, Kluyverweg 1, 2629 HS Delft, the Netherlands

2 Institute of Polymer Engineering, FHNW University of Applied Sciences and Arts Northwestern Switzerland, CH-5210, Windisch, Switzerland

*Corresponding authors

U.Farooq@tudelft.nl

C.A.Dransfeld@tudelft.nl

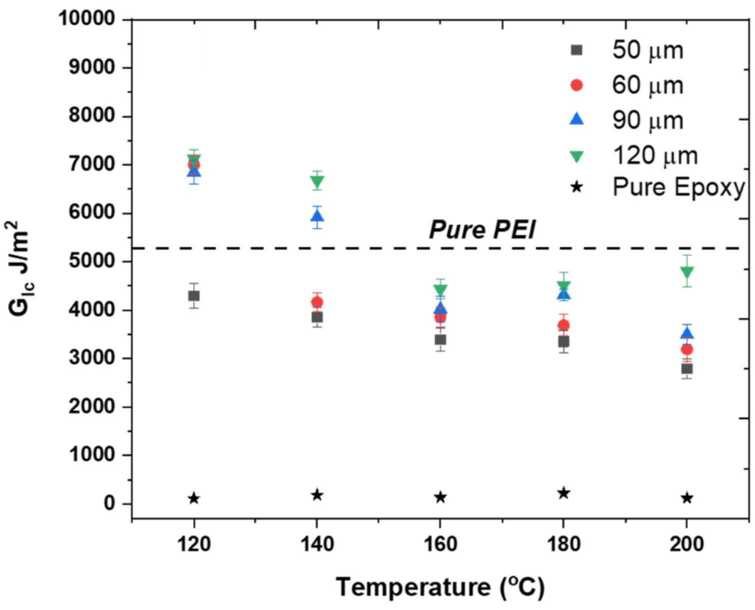

Figure S1. Energy release rate (G_{Ic}) as a function of cure temperature for different PEI layer thicknesses.

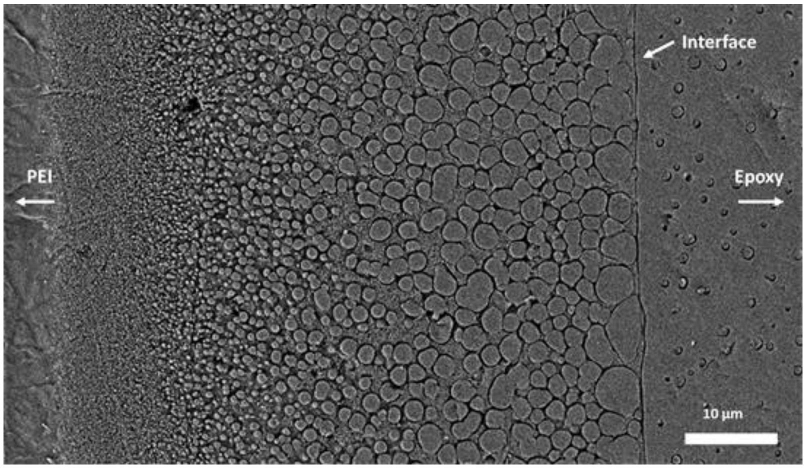

Figure S2. SEM micrograph showing phase-inverted morphology (epoxy-rich particles densely dispersed in a continuous PEI-rich matrix) of a PEI-epoxy interphase obtained at 180°C cure temperature.[1]

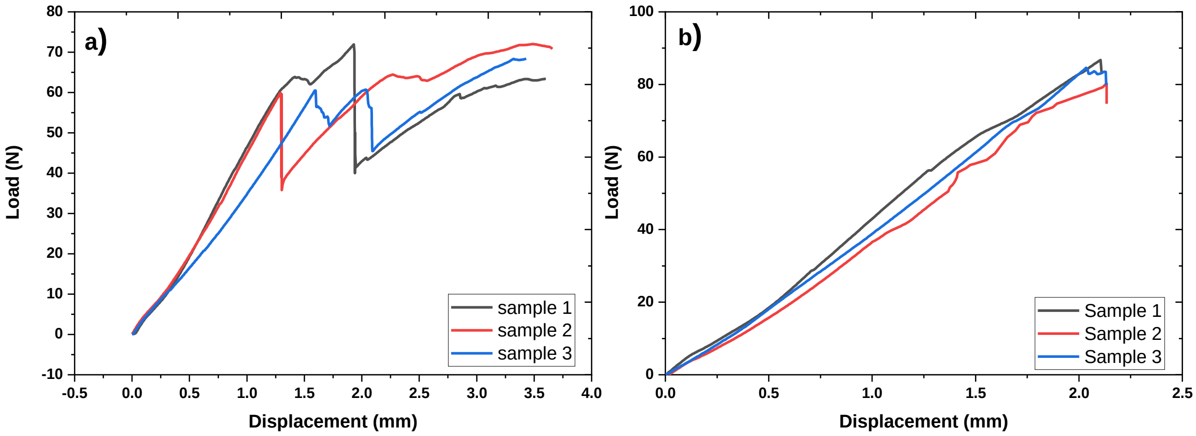

Figure S3. The load-displacement curves of three samples of PEI/epoxy system with 60 µm PEI layer, (a) cured at 120°C, and b) cured at 180°C.

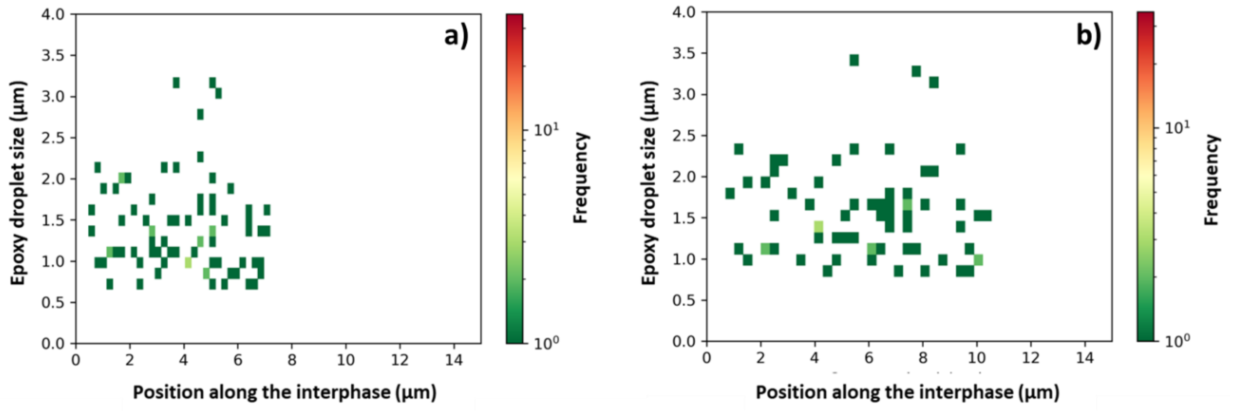

Figure S4. The epoxy droplet size from image analysis plotted as a function of position along the interphase (0 being pure EP) for samples with a) 90 µm PEI layer and b) 120 µm PEI layer, cured at 120ºC.

Calculation of plastic zone and critical defect size

Plastic zone size is calculated by Irwin model[2], under plane strain condition, written as follows:

$$Plastic\ zone\ size = \frac{1}{6\pi} \cdot \frac{k^2}{\sigma^2}$$

Critical defect size is calculated by Griffith model, under plane strain condition, written as follows:

$$Critical\ defect\ size = \frac{1}{\pi} \cdot \frac{k^2}{\sigma^2}$$

where k is the stress intensity factor with the value of 3.45 MPa.m$^{1/2}$ and σ is the yield strength of the material with the value of 105 MPa.

References

[1] Teuwen, J.; Asquier, J.; Inderkum, P.; Masania, K.; Brauner, C.; Villegas, I.; Dransfeld, C. Gradient Interphases Between High-T_g Epoxy And Polyetherimide For Advanced Joining Processes, In ECCM18: 18th European Conference On Composite Materials, 2018.

[2] Kolednik, O. Fracture Mechanics. In Wiley Encyclopedia of Composites; Nicolais, L., Ed.; 2012; pp 1-16.
